# Research on the equity of health resource allocation in TCM hospitals in China based on the Gini coefficient and agglomeration degree: 2009–2018

**DOI:** 10.1186/s12939-022-01749-7

**Published:** 2022-10-06

**Authors:** Guolin Dai, Ruifeng Li, Shuang Ma

**Affiliations:** 1 Institute of Medical Information, Chinese Academy of Medical Sciences & Peking Union Medical College, Beijing, 100020 China; 2 School of Management, Beijing University of Chinese Medicine, Beijing, 100029 China

**Keywords:** Health resources of TCM, Gini coefficient, Agglomeration degree, Equity

## Abstract

**Background:**

The demographical and geographical distributions of health resources are important aspects of healthcare access. Few studies have been published on health resource allocation in TCM hospitals in China despite public equity concerns.

**Methods:**

This article uses the Gini coefficient and agglomeration degree to analyze the health resources of TCM hospitals in China according to demographic and geographic configuration conditions in order to study the equity of the health resources of TCM from 2009 to 2018.

**Results:**

From 2009 to 2018, all regions of the TCM health resources per ten thousand people and per ten thousand square kilometers showed overall upward trends. The overall equity of the health resource allocations of TCM hospitals in China tended to improve year by year. However, there were still great differences among regions. Generally, the equity of physical resource allocation was better than the equity of human resource allocation. Additionally, the equity of health resources in TCM hospitals allocated by population was better than it was by geographic region.

**Conclusions:**

It is necessary to further optimize the structure of TCM resource allocation, and enhance the equity of resource allocation among different regions.

## Background

Traditional Chinese medicine (TCM) is a holistic system of medicine that has been part of Chinese culture for over 3000 years [1, 2]. TCM has been used for thousands of years in treating different types of diseases and symptoms, such as COVID-19 and other viral infections, allergic Diseases, dementia, cancer, geriatrics as well as mental illness [3–6]. Additionally, it is one of the oldest alternative healing options around the world and has gained increased application in many countries. Actually, China is the only country where both Western medicine and TCM are practiced alongside each other in hospitals and primary care facilities [2]. TCM has its own department, which is named the National Administration of Traditional Chinese Medicine. It also has branches at the provincial and county levels, as well as its own hospitals, medical schools, and research institutions [7]. The Chinese government has set out a series of measures to develop TCM, including advancing the historical place of TCM in health care, improving the ability to provide TCM services, and adjusting the allocation of health resources for TCM. Evidence shows that TCM can be recognized as one of the most important treatments in the world due to its unique advantages in health care [1, 8].

Although TCM is regarded as assuming a unique role in China’s medical service system, the proportions of its diagnosis and treatment volumes are not high. According to the data of the China Health Statistical Yearbook (2020), the total diagnosis and treatment volume of TCM reached 1163.9 million in 2019, accounting for 16.4% of the total diagnosis and treatment volume in China. TCM hospitals were the main Chinese medicine providers that treated patients with TCM services and products. In 2019, TCM hospitals were the core subjects providing TCM services, and the diagnosis and treatment volumes of TCM hospitals accounted for 58.02% of the total TCM diagnosis and treatment volume, while TCM clinics and outpatient departments accounted for the rest.

Along with the Chinese implementation of the national medical reform plan in 2009, the Chinese government has put emphasis on medical service allocation over the years [9–11]. Empirical work has shown that equitable allocation of health care resources ensures equity in health care accessibility, while inequalities in health care resources are associated with inequities in both health system quality and health outcomes [12, 13]. Actually, there are great differences in the economic situation between the eastern, central, and western region of China. Data showed that the total GDP of the eastern region accounted for 51.6% of the total national GDP in 2018, while the western region accounted for 22.5%. The per capita GDP in the Eastern, Central and Western regions accounted for 47.1, 27.3 and 25.6% of the total [14]. In contrast to the prosperous eastern region, and the industrial and agricultural central region, the western region, situated inland is environmentally and economically underdeveloped.

Similar to the geographical disparities in China’s economic development, regions in western and central China have much lower densities of health institutions, bed numbers and health workers than eastern China, which has more developed regions [15]. Despite the government’s support for TCM, TCM hospitals are no exception when it comes to these regional inequalities in the distribution of China’s health care resources, with the eastern region having higher bed counts and doctor densities than the western or central areas.

Although the TCM health service system in various regions of China has significantly improved since the 2009 reform plan, there have still been many problems regarding TCM health resources, such as their unreasonable allocation structure. TCM is an important aspect of Chinese health services, and with few studies published in English on the trends of TCM health resources in mainland China these years, some issues concerning equity still need to be explored. Therefore, this article aims to estimate the equity of TCM health resource allocation in mainland China from 2009 to 2018, and give some suggestions for optimizing TCM resource allocation.

## Methods

### Data resources and region division

**Fig. 1:**
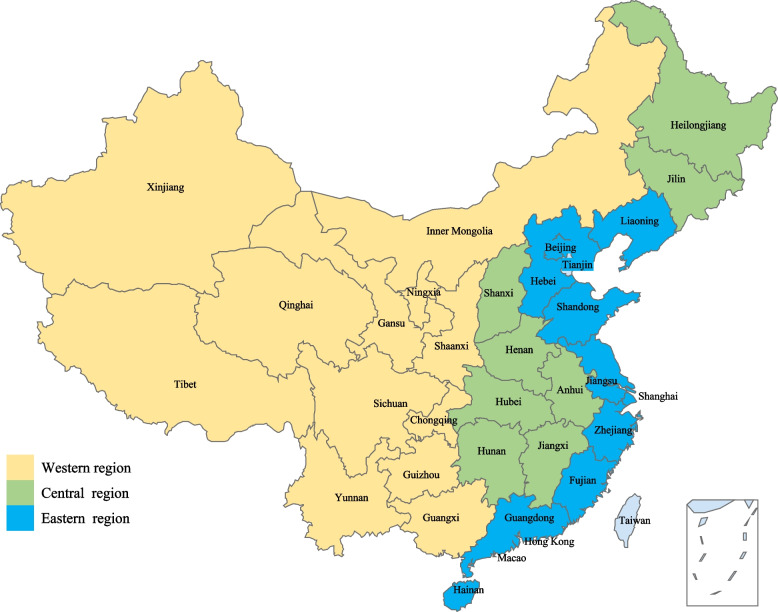
The division of eastern, central and western regions of China

In our study, the data were collected from the China Statistical Yearbook of TCM (2009–2018) and the China Health Statistical Yearbook (2010–2019), which covered 31 provinces, autonomous regions and municipalities. We excluded the regions of Hong Kong, Macao, and Taiwan because of the lack of relevant data [9]. In China, the eastern, central, and western regions were divided on the basis of geographical position and the economic development level, as shown in Fig. 1. The eastern region included Hebei, Liaoning, Jiangsu, Zhejiang, Fujian, Shandong, Guangdong, Hainan, Beijing, Tianjin, and Shanghai. The central region included Shanxi, Jilin, Heilongjiang, Anhui, Jiangxi, Henan, Hubei, Hunan. In addition, the western region included Sichuan, Guizhou, Yunnan, Shaanxi, Gansu, Qinghai, Inner Mongolia, Guangxi, Tibet, Ningxia, and Xinjiang and Chongqing.

### Indicators

Referring to previous studies, human resources and physical resources were considered important to the delivery of health services [16–18]. The indicators included institutions, bed numbers and health workers (practicing doctors, assistant practicing doctors, and technicians) in TCM hospitals in China. The number of institutions and beds represents the physical resources, and the health workers represent the human resources. These three indicators are commonly used to evaluate the equity of health resource allocation, and can reflect the overall health resource allocation situation [19, 20]. The average annual growth rate is also used to reflect average development trends.

The population and area data for each region came from the National Bureau of Statistics. This study used Excel to calculate the Gini coefficient and evaluate the equity of the health resource allocations of TCM hospitals in the eastern, central and western regions of China. They were examined from two aspects: the population distribution and the geographical distribution [21–23]. Additionally, the agglomeration degree was used to evaluate the equity of the health resource allocation of TCM hospitals.

### Gini coefficient

The Gini coefficient was developed based on the Lorenz curve [24], which is widely used to determine the equality of resource distribution [16]. In the formula, x_i_ refers to the cumulative population proportion, y_i_ refers to the cumulative proportion of health resources, n is the total number of provincial administrative region, i = 1, 2, … n. The Gini coefficient is between 0 and 1. The closer the value to 0, the more equitable the distribution, and vice versa. Different Gini values indicate different equality: ≤0.2 absolute equality, > 0.2 relative equality, 0 > 0.3 reasonably equal; > 0.4 relative inequality, > 0.5 serious inequality [25, 26]. G is calculated as follows:

$$G=1-{\sum}_{i=1}^n\left({x}_{i+1}-{x}_i\right)\left({y}_{i+1}+{y}_i\right)$$

### Health resource agglomeration degree

Chinese scholars Yuan S and others introduced the concept of the ‘agglomeration degree’ from the field of economics to the research into the equity of health resource allocation [27]. They put forward the concept of the health resource agglomeration degree (HRAD), a new index to evaluate the equity of health resource allocation. When using the HRAD to evaluate the equity of health resource allocation, we can take into account the influences of both population and geography at the same time, and measure the regional health resource allocation inequities between different groups [28]. The HRAD refers to the proportion (%) of health resources concentrated in the land area accounting for 1% of the upper level area in a certain region. The calculation formula is:

$$\textrm{HRADi}=\left[\left(\textrm{HRi}\div \textrm{HRn}\right)\times 100\%\right]\div \left[\left(\textrm{Ai}\div \textrm{An}\right)\times 100\%\right]=\left(\textrm{HRi}\div \textrm{Ai}\right)\div \left(\textrm{HRn}\div \textrm{An}\right)$$

HRADi represents the health resource agglomeration degree of region i, HRi is the number of health resources owned by region i, Ai is the land area of region i, HRn is the total health resources of the upper level region, and An is the total land area of the upper level region [29].

Generally, the equity of health resource allocation requires the consideration of two dimensions: namely, equity based on population distribution and accessibility based on geographical distribution. These two factors should be considered when evaluating the HRAD. The evaluation criteria of HRAD equity are: HRADi = 1or HRADi/PADi = 1. When HRADi = 1, it means that the distribution of health resources among different regions is absolutely fair according to the geographical scale; when HRADi/ PADi = 1, that means the distribution of health resources among different regions is absolutely fair according to the size of the population [30].

The Gini coefficient can only measure the equity of the overall resource allocation [31]. Therefore, this study also used the HRAD evaluation index, which comprehensively considers demographic and geographical factors, to measure the equity of the health resource allocation of TCM hospitals in different regions. In this study, the concept of HRAD is further embodied as the evaluation of the agglomeration degree of TCM institutions, bed numbers, health technicians and practicing (assistant) doctors.

## Results

### Basic situation of the health resource allocation of TCM hospitals nationwide

#### National health resource allocation of TCM hospitals in 2018

At the end of 2018, the total number of TCM hospitals nationwide reached 4939, including 1774 in the eastern region, 1483 in the central region and 1682 in the western region. The number of beds in TCM hospitals nationwide was 1021,548, the highest number being in the eastern region, accounting for 37.34%. The total number of health technicians in TCM hospitals nationwide reached 998,203, with 426,693 technicians in the eastern region, accounting for 42.75%. There were 174,596 TCM practicing (assistant) physicians, of which the eastern region had the highest number, 76,394, accounting for 43.75%.

**Fig. 2:**
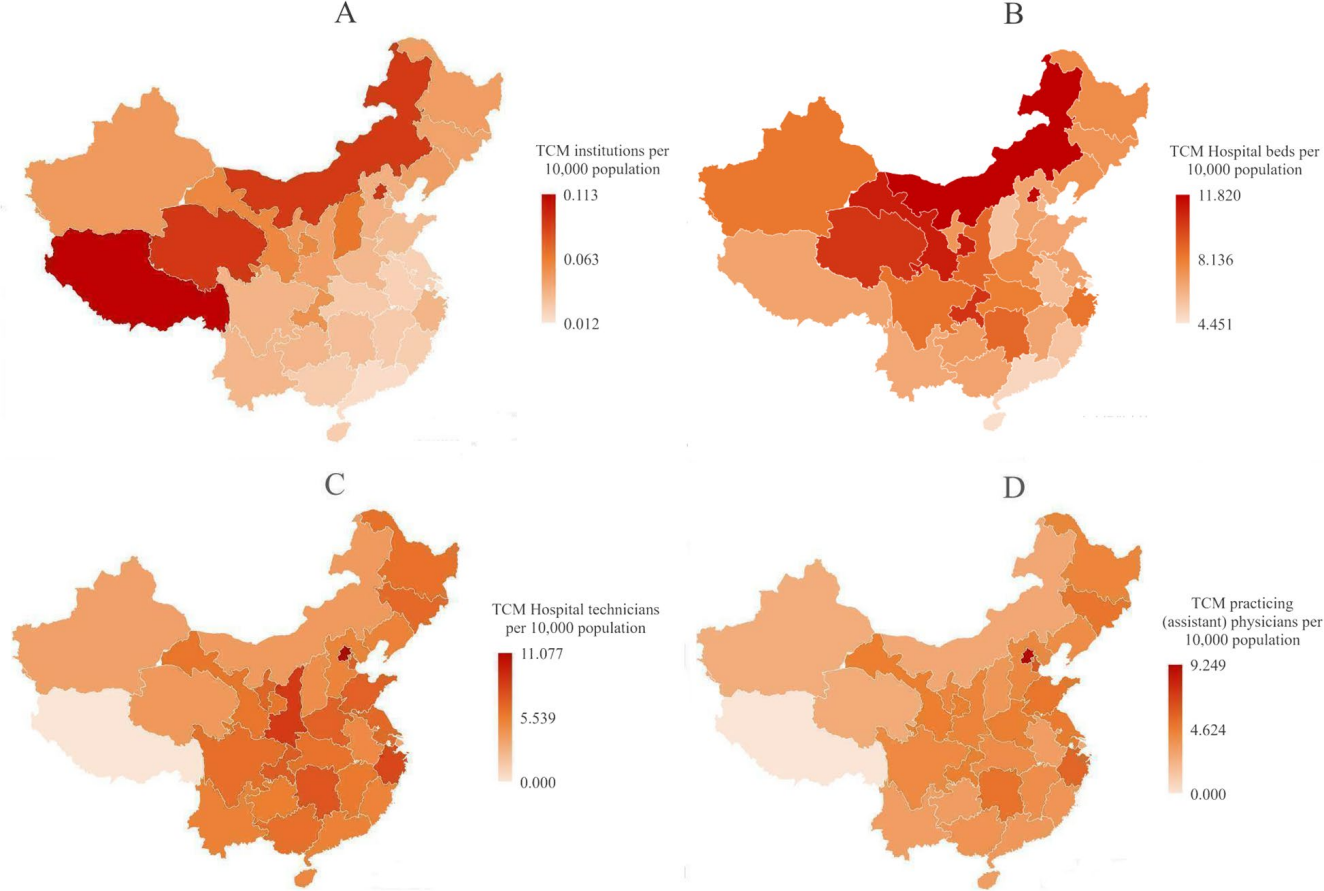
Distribution map of health resources of TCM hospitals per 10,000 people in 2018. **A** is the distribution map of the TCM institutions, **B** is the distribution map of the TCM Hospital beds, **C** is the distribution map of the TCM Hospital technicians, **D** is the distribution map of the TCM practicing (assistant) physicians

**Fig. 3:**
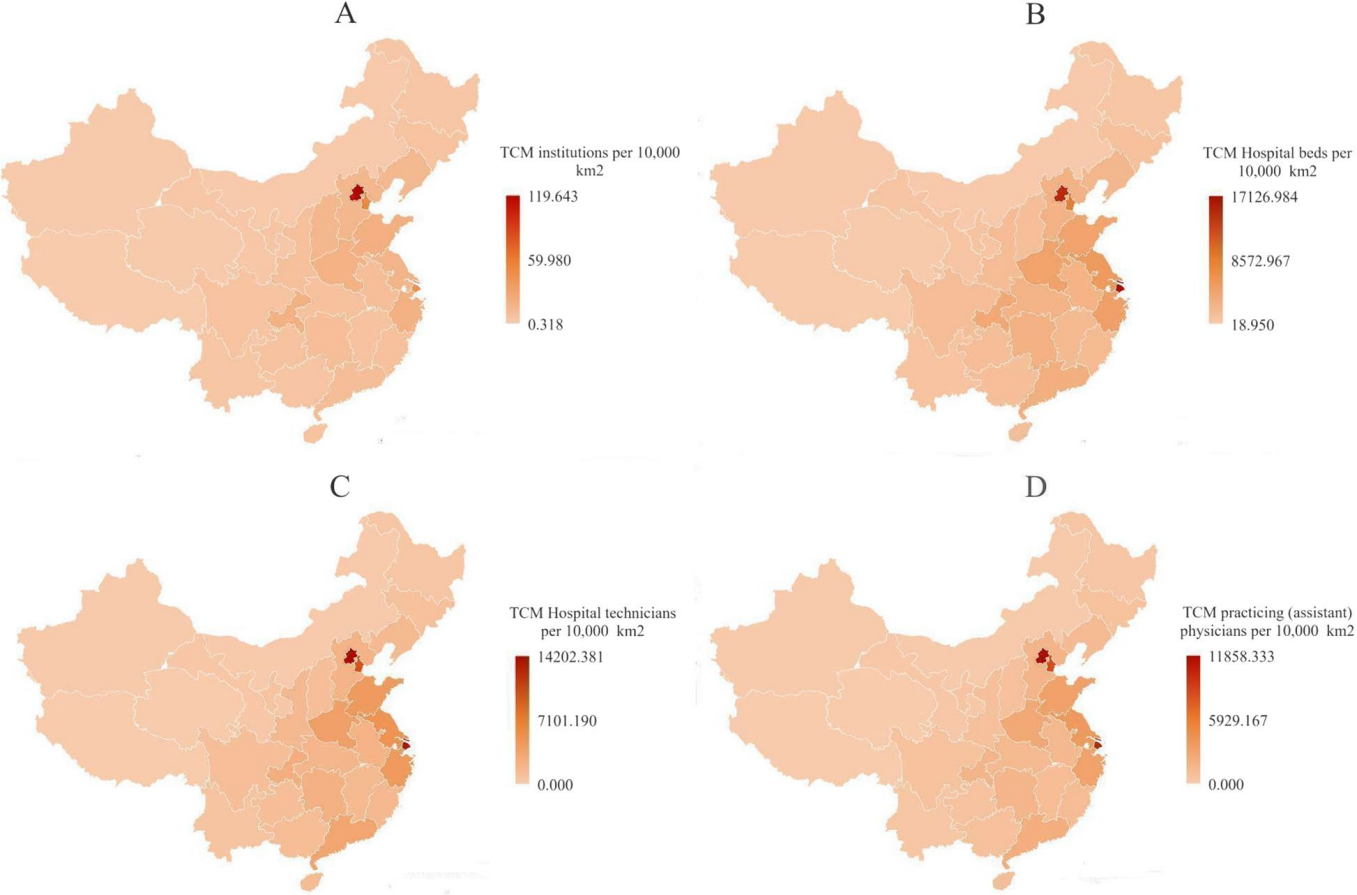
Distribution map of health resources of TCM hospitals per 10,000 km2 in 2018. **A** is the distribution map of the TCM institutions, **B** is the distribution map of the TCM Hospital beds, **C** is the distribution map of the TCM Hospital technicians, **D** is the distribution map of the TCM practicing (assistant) physicians

When considering the physical resource distribution of TCM hospitals nationwide by population, the western region had more health resources per 10,000 members of the population than the other two regions. And when considering their distribution by geographical area, the eastern and central regions had more health resources per 10,000 km^2^ than the western region. The same distribution characteristics were seen if the distribution of the human resources of TCM hospitals were considered by population. The geographic distributions of health resources per 10,000 members of the population and per 10,000 km^2^ by region nationwide at the end of 2018 are shown in Figs. 2 and 3.

#### Health resources of TCM hospitals from 2009 to 2018

Among all the four types of health resources, the eastern region had the largest total resources from 2009 to 2018. The TCM health resources per 10,000 people numbered higher in the western region than those in the other two regions, while the amount of TCM health resources per 10,000 km^2^ in the western region was the least among the three regions.

In terms of the number of TCM hospital institutions, the number of TCM hospital institutions in the eastern, central and western regions of the country generally showed a growing trend from 2009 to 2018, exhibiting the eastern region increasing from 1055 in 2009 to 1774 in 2018, with an average annual growth rate of 6.71%; in the same period, the number in the central region increased from 1072 to 1483, with an average annual growth rate of 4.14%, and number in the western region increased from 1037 to 1682, with an average annual growth rate of 6.23%. The numbers of resources per 10,000 people and per 10,000 km^2^ were in a stage of rapid growth. In terms of the number of TCM hospital beds, the number of TCM hospital beds in the eastern, central and western regions all showed steady increasing trends; among the changes in resource ownership per 10,000 people, the western region had the fastest growth rate, with average annual growth of 13.17%, and the resource ownership per 10,000 km^2^ increased in all three regions, with the western region exhibiting the highest average annual growth rate of 13.65%. In terms of TCM practicing (assistant) physicians, the number of TCM practicing (assistant) physicians in the eastern, central and western regions all increased; resource ownership per 10,000 people was the highest in the eastern region until 2016, as in 2017 the western region surpassed the eastern region at 6.88 per 10,000 people. Resource ownership per 10,000 km^2^ in all three regions showed an increasing trend, but the average annual growth rate in the central region was less than 10%. In terms of the number of health technicians in TCM hospitals, all three regions exhibited upward trends. In terms of the total amount of resources, the central and western regions had significantly fewer resources than the eastern region. The number of resources per 10,000 people was still the largest and fastest growing in the western region, but there was a large gap with the eastern and central regions regarding the number of resources per 10,000 km^2^. The specific changes in resources per 10,000 people and per 10,000 km^2^ in each region are shown in Tables 1 and 2.

**Table 1:** TCM hospitals health resources per 10,000 people from 2009 to 2018

Year	physical resources						human resources					
	Institutions per 10,000 people			beds per 10,000 people			TCM practicing (assistant) physicians per 10,000 people			TCM health technicians per 10,000 people		
	Eastern	Central	Western	Eastern	Central	Western	Eastern	Central	Western	Eastern	Central	Western
2009	0.02	0.03	0.03	3.34	3.22	3.13	3.98	3.58	3.02	2.10	1.90	2.22
2010	0.02	0.03	0.03	3.55	3.51	3.54	4.15	3.81	3.38	2.21	2.02	2.42
2011	0.02	0.03	0.03	3.86	3.94	4.10	4.41	4.04	3.70	2.34	2.07	2.54
2012	0.02	0.03	0.03	4.37	4.50	4.87	4.82	4.42	4.22	2.70	2.33	2.94
2013	0.02	0.03	0.03	4.78	5.00	5.58	5.25	4.78	4.42	2.90	2.45	3.12
2014	0.02	0.03	0.03	5.16	5.47	6.22	5.49	5.03	5.13	3.10	2.69	3.47
2015	0.02	0.03	0.04	5.52	5.94	6.66	6.03	5.46	5.69	3.36	2.85	3.68
2016	0.03	0.03	0.04	5.82	6.34	7.21	6.37	5.86	6.33	3.57	3.00	3.93
2017	0.03	0.03	0.04	6.23	6.81	7.82	6.78	6.24	6.88	3.92	3.24	4.23
2018	0.03	0.03	0.04	5.97	7.35	8.42	6.86	6.61	7.45	4.19	3.44	4.36
Average annual growth rate %	**5.20**	**0.00**	**3.66**	**7.53**	**10.87**	**13.17**	**7.04**	**7.97**	**11.95**	**9.02**	**7.70**	**8.80**

**Table 2:** TCM hospitals health resources per 10,000 km^2^ from 2009 to 2018

Year	physical resources						human resources					
	Institutions per 10,000 km^2^			beds per 10,000 km^2^			TCM practicing (assistant) physicians per 10,000 km^2^			TCM health technicians per 10,000 km^2^		
	Eastern	Central	Western	Eastern	Central	Western	Eastern	Central	Western	Eastern	Central	Western
2009	9.94	6.35	1.51	1659.01	805.50	166.92	1045.89	473.67	118.59	1975.90	893.37	161.10
2010	10.40	6.35	1.54	1634.61	836.48	185.72	1143.11	506.19	126.90	2151.07	953.30	177.21
2011	10.74	6.46	1.57	2013.67	988.64	216.13	2305.11	1012.99	195.01	1222.75	518.44	133.65
2012	11.16	6.55	1.63	2296.37	1133.87	258.13	1419.85	586.36	155.62	2537.82	1113.06	223.49
2013	11.87	6.75	1.73	2532.01	1264.07	297.41	984.35	965.29	166.05	2777.20	1208.91	250.69
2014	12.33	6.92	1.82	2749.18	1387.34	332.87	1654.11	682.72	185.65	2997.00	1292.86	281.03
2015	13.09	7.27	1.96	2957.66	1513.69	363.25	1802.15	726.83	200.85	3233.50	1391.82	310.06
2016	14.02	7.73	2.10	3140.58	1623.91	392.15	1928.44	768.20	213.95	3439.44	1500.62	344.31
2017	15.59	8.08	2.25	3399.64	1751.88	428.40	2135.82	834.24	231.80	3685.41	1605.36	377.21
2018	16.71	8.79	2.45	3602.09	1898.22	464.73	2719.54	996.91	269.89	4018.96	1708.37	411.59
Average annual growth rate %	**6.71**	**4.15**	**6.24**	**10.18**	**11.31**	**13.65**	**12.69**	**9.75**	**10.83**	**9.28**	**8.44**	**12.44**

### Analysis of the changes in the equity of health resources in TCM hospitals nationwide

Based on the population, area and health resource data of the eastern, central and western regions from 2009 to 2018, the Gini coefficient of the population equity and the geographic equity of health resources of TCM hospitals were calculated, and the results are shown in Tables 3 and 4.

**Table 3:** Gini coefficient of TCM health resources by population trend from 2009 to 2018

Year	physical resources						human resources					
	Institutions			beds			TCM health technicians			TCM practicing (assistant) physicians		
	Eastern	Central	Western	Eastern	Central	Western	Eastern	Central	Western	Eastern	Central	Western
2009	0.1866	0.1853	0.2237	0.0919	0.1044	0.0973	0.1042	0.1212	0.0776	0.1305	0.1879	0.1165
2010	0.2198	0.1736	0.2152	0.0954	0.0972	0.0920	0.0986	0.1204	0.0707	0.1199	0.1738	0.1100
2011	0.2184	0.1783	0.1965	0.0925	0.0920	0.0947	0.0925	0.0947	0.0920	0.1248	0.1780	0.1048
2012	0.2236	0.1766	0.1900	0.1014	0.0886	0.0742	0.1102	0.1246	0.0700	0.1091	0.1769	0.1049
2013	0.2215	0.1708	0.1814	0.1048	0.0734	0.0656	0.1162	0.1070	0.0645	0.1030	0.1698	0.1024
2014	0.2197	0.1825	0.1726	0.1075	0.0773	0.0682	0.1232	0.1022	0.0636	0.1039	0.1912	0.0996
2015	0.2319	0.1758	0.1721	0.1051	0.0803	0.0716	0.1261	0.1006	0.0681	0.1113	0.1935	0.1015
2016	0.2402	0.1684	0.1755	0.1083	0.1803	0.1704	0.1252	0.0814	0.1019	0.1073	0.1785	0.0869
2017	0.2384	0.1734	0.1806	0.1119	0.1780	0.1918	0.1259	0.0664	0.0937	0.1079	0.1672	0.1044
2018	0.2376	0.1719	0.1862	0.1204	0.1738	0.1967	0.1264	0.0602	0.0943	0.1085	0.1693	0.1036

**Table 4:** Gini coefficient of TCM health resources by geographical region trend from 2009 to 2018

Year	physical resources						human resources					
	Institutions			beds			TCM health technicians			TCM practicing (assistant) physicians		
	Eastern	Central	Western	Eastern	Central	Western	Eastern	Central	Western	Eastern	Central	Western
2009	0.1831	0.5504	0.2898	0.2601	0.6098	0.3128	0.2875	0.6356	0.3035	0.2653	0.6580	0.3177
2010	0.1994	0.5460	0.2896	0.3466	0.6091	0.3348	0.2901	0.6427	0.3023	0.2616	0.6557	0.3089
2011	0.2207	0.5445	0.2901	0.2538	0.6090	0.3276	0.2920	0.6451	0.3064	0.2680	0.6531	0.3077
2012	0.2336	0.5395	0.2817	0.2630	0.6195	0.3361	0.3000	0.6551	0.3194	0.2640	0.6551	0.3089
2013	0.2514	0.5483	0.2921	0.2684	0.6192	0.3374	0.3092	0.6505	0.3287	0.2706	0.6443	0.3199
2014	0.2603	0.5393	0.2961	0.2672	0.6158	0.3405	0.3158	0.6514	0.3364	0.2721	0.6546	0.3276
2015	0.2677	0.5400	0.2963	0.2660	0.6139	0.3405	0.3180	0.6473	0.3392	0.2777	0.6526	0.3310
2016	0.2677	0.5402	0.3095	0.2723	0.6180	0.3423	0.3205	0.6408	0.3382	0.2797	0.6491	0.3357
2017	0.2827	0.5442	0.2988	0.2659	0.5882	0.3417	0.3182	0.6412	0.3428	0.2766	0.6461	0.3412
2018	0.2846	0.5467	0.2968	0.2643	0.5898	0.3420	0.3194	0.6426	0.3473	0.2789	0.6443	0.3428

#### The Gini coefficient of TCM hospital health resources by population trend from 2009 to 2018

**Fig. 4:**
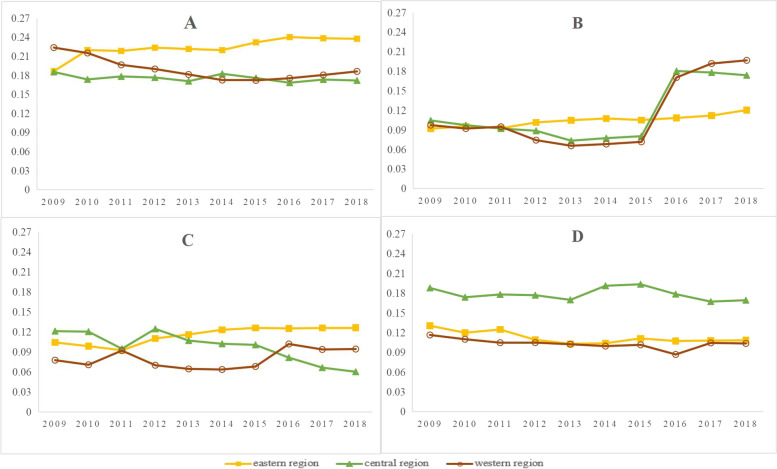
The Gini coefficient of the equity of the distribution of health resources by population in TCM hospitals from 2009 to 2018. **A** is the Lorenz curves of the institutions, **B** is the Lorenz curves of the TCM Hospital beds, **C** is the Lorenz curves of the TCM Hospital technicians, **D** is the Lorenz curves of the TCM practicing (assistant) physicians

The Gini coefficients of the distribution of physical and human health resources by population in TCM hospitals in all years were less than 0.3, indicating the equity of the distribution of health resources in TCM hospitals by population was good, as shown in Table 3. The trends of the Gini coefficient of the equity of the distribution of health resources by population in TCM hospitals from 2009 to 2018 are shown in Fig. 4.

The longitudinal analysis shows: 1) In the eastern region, the Gini coefficient of the population allocation showed an overall weak and fluctuating upward trend from 2009 to 2018, from 0.1866 to 0.2376 for TCM hospitals, from 0.0919 to 0.1204 for beds in TCM hospitals, from 0.1042 to 0.1264 for health technicians in TCM hospitals, and from 0.1305 to 0.1085 for practicing (assistant) doctors of TCM. 2) In the central region: Gini coefficient allocated by population showed a slightly weak downward trend from 2009 to 2018, approximately 0.1853 for TCM hospitals, and approximately 0.1719 for beds in TCM hospitals; the Gini coefficient of health technicians dropped from 0.1044 to 0.0734, after rising to 0.1738. The Gini coefficient of health technicians dropped from 0.1212 to 0.0602, and the Gini coefficient of practicing (assistant) doctors was significantly lower in 2014 and 2015. 3) In the western region: the Gini coefficient allocated by population basically showed a weak upward trend from 2009 to 2018, moving from 0.2237 to 0.1862 for TCM hospitals, from 0.0973 to 0.1967 for beds in TCM hospitals, from 0.0776 to 0.0943 for health technicians in TCM hospitals, and was approximately 0.1 for practicing (assistant) doctors of TCM.

#### Changes in the Gini coefficients of TCM hospital health resources by geographical region trends from 2009 to 2018

**Fig. 5:**
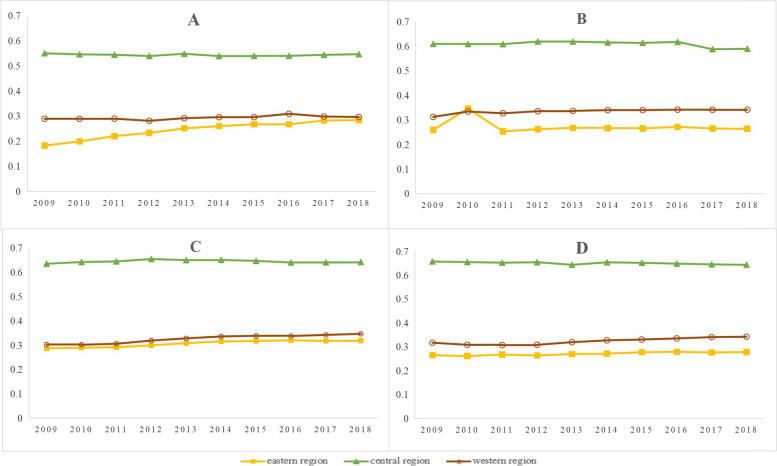
The Gini coefficient of equity in the geographical distribution of health resources in TCM hospitals from 2009 to 2018. **A** is the Lorenz curves of the institutions, **B** is the Lorenz curves of the TCM Hospital beds, **C** is the Lorenz curves of the TCM Hospital technicians, **D** is the Lorenz curves of the TCM practicing (assistant) physicians

The Gini coefficients of the distributions of physical and human health resources by population in TCM hospitals over the years are shown in Table 4. The trends of the Gini coefficients of equity in the geographical distribution of health resources in TCM hospitals from 2009 to 2018 are shown in Fig. 5.

Longitudinal analysis that: 1) In the eastern region, the geographically distributed Gini coefficient showed an overall weak and fluctuating upward trend from 2009 to 2018, moving from 0.1831 to 0.2827 for TCM hospitals, being approximately 0.26 for beds in TCM hospitals, and reaching 0.3466 in 2010; it moved from 0.2875 to 0.3182 for health technicians in TCM hospitals, and from 0.2653 to 0.2766 for practicing (assistant) doctors of TCM. 2) In the central region, the geographically distributed Gini coefficient showed an overall slightly stable trend from 2009 to 2018, being approximately 0.54 for TCM hospitals, approximately 0.61 for beds in TCM hospitals; moving from 0.6356 to 0.6514 and back to 0.6412 for health technicians in TCM hospitals, and moving from 0.6580 to 0.6416 for practicing (assistant) doctors of TCM. The Gini coefficients of various health resources exceeded the warning lines and were in a highly inequitable state from 2009 to 2017, during which time human health resources were the most unfairly allocated. 3) In the western region, the geographically distributed Gini coefficient basically showed a stable trend from 2009 to 2018, and the medical material resources of TCM basically showed a stable trend. Health technicians in TCM hospitals and practicing (assistant) doctors of TCM displayed a slight upward trend, moving from 0.3035 to 0.3428 for the former and from 0.3177 to 0.3412 for the latter.

### Analysis of the health resource aggregation of TCM hospitals Nationwide

#### Analysis of the health resource aggregation TCM hospitals Nationwide in 2018

##### Geographical aggregation

As shown in Table 5, the agglomeration degree of the four indexes of the health resources of TCM hospitals in the provinces of Inner Mongolia, Heilongjiang, Yunnan, Xizang, Gansu, Qinghai, Ningxia, and Xinjiang was less than 1, and most of these provinces were distributed in the western region. The agglomeration degree of practicing (assistant) doctors of TCM in other provinces was basically greater than l, which indicated the health resources of TCM hospitals in other provinces were fairly allocated according to region compared with the abovementioned provinces. Among them, the agglomeration degree of the health resources of TCM hospitals in Beijing and Shanghai provinces ranked first, the agglomeration degrees of TCM hospitals in Tibet were the smallest, and the agglomeration degree of the health resources of TCM hospitals in Beijing were more than five times higher than the national average, indicating that the health resources of TCM hospitals were too agglomerated in such provinces.

**Table 5:** Concentration of Health Resources and Population Aggregation of TCM Hospitals in China in 2018

Region	Population Aggregation	Institutions		TCM Hospital beds		TCM health technicians		TCM practicing (assistant) physicians	
		HRAD	Ratio	HRAD	Ratio	HRAD	Ratio	HRAD	Ratio
**Eastern region**	3.990	3.257	0.816	3.395	0.851	3.877	0.972	3.968	0.994
**Central region**	1.707	1.713	1.004	1.789	1.048	1.648	0.965	1.637	0.959
**Western region**	0.365	0.477	1.307	0.438	1.200	0.397	1.088	0.385	1.055
Beijing	8.840	23.324	2.639	14.512	1.642	21.100	2.387	30.341	3.432
Tianjin	9.518	10.006	1.051	8.045	0.845	10.482	1.101	14.729	1.547
Hebei	2.775	2.950	1.063	2.579	0.929	2.445	0.881	2.459	0.886
Shanxi	1.640	3.018	1.840	1.269	0.774	1.227	0.748	1.339	0.817
Inner Mongolia	0.148	0.376	2.544	0.239	1.616	0.222	1.506	0.243	1.643
Liaoning	2.060	2.699	1.310	2.074	1.007	1.701	0.826	1.866	0.906
Jilin	0.995	1.259	1.265	0.995	1.000	1.021	1.026	1.263	1.270
Heilongjiang	0.550	0.730	1.326	0.568	1.032	0.488	0.888	0.493	0.896
Shanghai	26.528	8.974	0.338	16.143	0.609	22.224	0.838	26.541	1.000
Jiangsu	5.410	3.325	0.615	5.044	0.932	5.704	1.054	5.441	1.006
Zhejiang	3.878	3.899	1.005	4.428	1.142	6.068	1.565	4.498	1.160
Anhui	3.121	1.912	0.613	2.535	0.812	2.357	0.755	2.267	0.726
Fujian	2.240	1.479	0.660	1.719	0.767	1.903	0.849	1.956	0.873
Jiangxi	1.919	1.366	0.712	1.772	0.923	1.662	0.866	1.472	0.767
Shandong	4.504	4.094	0.909	4.106	0.912	4.584	1.018	4.452	0.988
Henan	3.965	3.817	0.963	4.181	1.054	4.030	1.016	4.134	1.043
Hubei	2.194	1.552	0.707	2.418	1.102	2.172	0.990	1.804	0.822
Hunan	2.246	1.970	0.877	2.688	1.197	2.418	1.077	2.345	1.044
Guangdong	4.346	1.993	0.459	2.952	0.679	3.598	0.828	3.612	0.831
Guangxi	1.439	0.983	0.683	1.335	0.927	1.550	1.077	1.416	0.984
Hainan	1.894	1.261	0.666	1.208	0.638	1.463	0.772	1.371	0.724
Chongqing	2.599	3.861	1.486	3.651	1.405	3.076	1.184	2.543	0.979
Sichuan	1.195	1.215	1.017	1.377	1.152	1.222	1.023	1.152	0.964
Guizhou	1.410	1.396	0.990	1.362	0.966	1.275	0.904	1.194	0.847
Yunnan	0.869	0.860	0.989	0.783	0.901	0.697	0.802	0.639	0.736
Tibet	0.019	0.062	3.206	0.018	0.925	0.015	0.758	0.041	2.104
Shaanxi	1.296	1.678	1.295	1.554	1.199	1.676	1.294	1.091	0.842
Gansu	0.400	0.652	1.630	0.600	1.499	0.390	0.976	0.486	1.214
Qinghai	0.058	0.148	2.579	0.080	1.398	0.062	1.072	0.079	1.369
Ningxia	0.714	0.969	1.356	0.705	0.987	0.730	1.022	0.762	1.067
Xinjiang	0.103	0.142	1.376	0.117	1.131	0.099	0.954	0.126	1.220

##### Population aggregation

There were significant differences in the ratios of the agglomeration degrees of the health resources of TCM hospitals. The ratios of the agglomeration degrees of the health material resources of TCM hospitals and the agglomeration degrees of the populations in the central and western regions were closer to 1 than those of the eastern region, and the ratios of the agglomeration degrees of the human health resources of TCM hospitals and the agglomeration degrees for the populations in the three regions were close to 1. Among them, the TCM hospitals in Zhejiang, Henan, Yunnan, and Guizhou were the best and most fairly allocated according to the population, and the number of beds in TCM hospitals in Beijing had been allocated in a way that was more concentrated than the population; in Shanghai, the ratio of the agglomeration degree of practicing (assistant) doctors and agglomeration degree of the population was 1, which was most fairly allocated; in Jiangsu and Shandong, the ratios of the agglomeration degree and agglomeration degree of the population were close to 1, which were relatively fairly allocated, and the practicing (assistant) doctors of TCM in Hainan and Yunnan provinces were relatively insufficient.

#### Analysis of the health resources in TCM hospitals from 2009 to 2018

As shown in Table 6, the agglomeration degree of the health resources of the TCM hospitals among in the three regions of China was only less than 1 in the western region from 2009 to 2018, which indicates the health resources of the TCM hospitals in the western region were poorly allocated according to the geographical scale; the agglomeration degree of population in the eastern region of China showed an upward trend, and the agglomeration degree of the health resources of TCM hospitals allocated by geographical area and population exhibited a downward trend; both indicators in the central region showed upward trends, and the ratio was closer to 1 from 2009 to 2018, which indicates that the equity of TCM hospitals in the central region allocated by population improved; the agglomeration degree of the TCM hospitals in the western region according to the geographical area varied slightly, and was far less than 1, with the ratio of the agglomeration degree of the population beginning to decline from the year 2014, and the accessibility of general practitioners in the western region being poor.

**Table 6:** HRAD and Population Aggregation of TCM Hospitals in China from 2009 to 2018

Year	Region	Population Aggregation	Institutions		TCM Hospital beds		TCM health technicians		TCM practicing (assistant) physicians	
			HRAD	Ratio	HRAD	Ratio	HRAD	Ratio	HRAD	Ratio
2009	Eastern region	3.634	3.024	0.832	1.030	0.283	4.036	1.110	3.694	1.017
	Central region	1.827	1.932	1.058	0.994	0.544	1.825	0.999	1.673	0.916
	Western region	0.390	0.459	1.175	0.964	2.469	0.329	0.843	0.419	1.073
2010	Eastern region	3.742	3.098	0.828	1.005	0.269	4.052	1.083	3.742	1.000
	Central region	1.808	1.892	1.046	0.992	0.548	1.796	0.993	1.657	0.917
	Western region	0.378	0.457	1.208	1.002	2.649	0.334	0.882	0.415	1.098
2011	Eastern region	3.751	3.125	0.833	0.976	0.260	4.036	1.076	3.807	1.015
	Central region	1.803	1.879	1.042	0.997	0.553	1.774	0.984	1.614	0.895
	Western region	0.378	0.456	1.206	1.039	2.748	0.341	0.903	0.416	1.100
2012	Eastern region	3.758	3.152	0.839	0.960	0.256	3.999	1.064	4.063	1.081
	Central region	1.799	1.849	1.028	0.990	0.551	1.754	0.975	1.730	0.962
	Western region	0.378	0.459	1.215	1.072	2.834	0.352	0.931	0.348	0.921
2013	Eastern region	3.762	3.183	0.846	0.944	0.251	3.954	1.051	4.248	1.129
	Central region	1.796	1.811	1.008	0.987	0.549	1.706	0.950	1.966	1.094
	Western region	0.378	0.464	1.226	1.102	2.912	0.371	0.980	0.262	0.691
2014	Eastern region	3.765	3.181	0.845	0.931	0.247	3.954	1.050	3.983	1.058
	Central region	1.794	1.787	0.996	0.986	0.550	1.706	0.951	1.727	0.963
	Western region	0.378	0.470	1.243	1.122	2.963	0.371	0.980	0.361	0.955
2015	Eastern region	3.754	3.178	0.847	0.926	0.247	3.933	1.048	3.984	1.061
	Central region	1.786	1.766	0.989	0.996	0.557	1.693	0.948	1.702	0.953
	Western region	0.382	0.476	1.246	1.118	2.926	0.377	0.988	0.367	0.961
2016	Eastern region	3.743	3.184	0.851	0.921	0.246	3.872	1.034	3.953	1.056
	Central region	1.813	1.756	0.969	0.983	0.542	1.689	0.932	1.688	0.931
	Western region	0.377	0.477	1.266	1.141	3.027	0.388	1.028	0.375	0.996
2017	Eastern region	3.778	3.287	0.870	0.911	0.241	3.850	1.019	3.966	1.050
	Central region	1.781	1.704	0.957	0.996	0.559	1.677	0.942	1.669	0.937
	Western region	0.380	0.474	1.249	1.142	3.009	0.394	1.038	0.378	0.996
2018	Eastern region	3.990	3.257	0.816	3.395	0.851	3.877	0.971	3.968	0.994
	Central region	1.707	1.713	1.003	1.789	1.048	1.648	0.965	1.637	0.959
	Western region	0.365	0.477	1.307	0.438	1.201	0.397	1.088	0.385	1.057

## Discussions

This article analyzed the data of health resources in TCM hospital from 31 provinces in China from 2009 to 2018. Based on the population and geographical allocation, we discussed the equity of the distributions of health resources by the Gini coefficient and the agglomeration degree. Our study had three major findings.

First, there were obvious regional differences in the allocation of health resources in TCM hospitals in China. The total amount of the various health resources of TCM hospitals in China showed an upward trend from 2009 to 2018. However, there were obvious regional differences in the allocations of health resources in TCM hospitals in China as a whole, and there was regional differentiation in the structural layouts of health resources in TCM hospitals. At the geographical level, the health resources of TCM hospitals in China were mainly concentrated in the economically developed eastern region, while the health resources of TCM hospitals in the relatively underdeveloped western region were obviously insufficient, especially considering the obvious gap in terms of the health technicians of TCM hospitals and the practicing (assistant) doctors of TCM, and there was a large gap in the allocation of the health resources of TCM hospitals among regions. At the population level, per capita resources in western regions such as Xizang, Inner Mongolia and Gansu were more abundant and had grown the fastest since the new medical reforms. Due to the relatively small populations and relatively large per capita health resources in the western region, as well as the influence of policy preference, there had been an increase in the investment in health resources in the western region, which promoted the rapid growth of TCM health resources. From the perspective of the degree of regional agglomeration, there were obvious differences in the degrees of agglomeration of the health resources of TCM hospitals in the eastern, central and western regions of China. The agglomeration degrees of various TCM hospital health resources in Beijing, Shanghai, and Tianjin in the eastern region were significantly greater than 1, as the health resources of TCM hospitals were too concentrated, while the agglomeration degrees of TCM hospitals of Xinjiang, Xizang, Gansu, Qinghai, Ningxia in the western region were less than 1. These results were consistent with the research results of concerning overall health resource allocation [9, 32, 33]. Additionally, the differences between different regions were also closely related to the degrees of the regions’ economic development [34]. Based on this, the government should support areas with insufficient health resources in TCM hospitals in the western region, and shorten the TCM health resource allocation gaps in the allocation of health resources of TCM between regions in the future.

Second, the equity of the allocation of physical health resources was better than that of the allocation of human resources in TCM hospitals. According to the Gini coefficient data from 2009 to 2018, the equity of allocation of the health material resources of TCM hospitals according to geographical area was better than that of the human health resources of TCM hospitals. It was found through a horizontal comparison that the Gini coefficients for human health resources of TCM hospitals were greater than those of the material resources in each year. In particular, the human health resources in TCM hospitals in the western region were highly unfairly allocated, and the impact of the unfair allocation of human resource indicators in TCM hospitals was relatively huge, which indicated that the human health resources in TCM hospitals were inequitably allocated compared with the physical health resources in TCM hospitals. This might be closely related to the structure of the economic development of various regions in China. Although the physical health resources of TCM hospitals in various regions were sufficiently allocated, the human resources for TCM, such as students majoring in TCM and medical personnel, preferred to choose the eastern region, which features relatively richer resources and a more developed economy. As a result, most TCM human health resources were concentrated in the eastern region, while there was a lack of human resources in TCM hospitals in the central and western regions. This was consistent with the research conclusion reached by Fu that human health resources of TCM in China were poorly allocated [35]. And another reason for this may be that the physical resources such as medical institutions and beds are mainly implemented under the guidance of the Chinese government, while health professionals are often attracted by monetary incentives and opportunities for career progression. Well-developed areas provide higher salaries and better opportunities [10].

Third, the equity of the demographical distribution of health resources in TCM hospitals is better than that of the geographical distribution.

For the four indicators of TCM hospitals, the beds of TCM hospitals, the health technicians of TCM hospitals and the practicing (assistant) doctors of TCM, the Gini coefficients of the health resources of TCM hospitals in China allocated by population were below 0.3, the distribution of TCM hospital health resources per 10,000 people were equitable across different regions from 2009 to 2018. A large gap existed in the distribution inequality of TCM hospital health resources per square kilometer across different regions. Specially, the Gini coefficients for health resources allocated according to geography in various TCM hospitals in the central region was above 0.5, which was in an unfair state. This result indicated that the population allocation of health resources in TCM hospitals is better, which was consistent with the conclusions of other research [36–38]. This might be because health resources and regional health planning have been allocated according to populations for a long time, with the possession of health resources per 1000 people taken as the measurement index and less consideration being given to factors such as the geographical accessibility. Therefore, the government should increase the allocation of the health resources of TCM in economically backward areas and improve accessibility to health resources in remote areas. In the allocation of health resources in TCM hospitals, administrative health departments should not only consider demographic and economic factors, but also properly consider geographical area factors and regional accessibility for the utilization of health resources in TCM.

Several limitations of our study should be born in mind. Although the selection of indicators was based on the published literatures, other indicators associated with health resources allocation, such as the number of diagnostic equipment, were not investigated in this study. Future studies should consider additional indicators to have a comprehensive evaluation. Also, the analysis of different provinces was relatively inadequate, and additional research can focus on various provinces in the future.

## Conclusions

From 2009 to 2018, TCM health resources per ten thousand members of the population and per ten thousand square kilometers showed overall upward trends in all regions. The overall equity of the health resource allocation of TCM hospitals in China tended to improve year by year. However, there are still great differences among regions. Generally, the equity of physical resource allocation is better than that of human resource allocation. Additionally, the population allocation of health resources in TCM hospitals in China is better than the geographical distribution. Therefore, a targeted health policy with more equitable TCM resource allocation should be a priority for China’s healthcare system reforms.

## Data Availability

The data were collected from the China Statistical Yearbook of TCM (2009–2018) and the China Health Statistical Yearbook (2010–2019), which were published by the National Health Commission of China. The datasets generated by the authors are available from the corresponding author on reasonable request.
